# Supplementary material for: Deep learning enables structured illumination microscopy with low light levels and enhanced speed
Source: Nat Commun. 2020 Apr 22;11:1934. doi: 10.1038/s41467-020-15784-x (PMC7176720; doi:10.1038/s41467-020-15784-x)
Supplement: Supplementary file 1 — Supplementary Information [file 41467_2020_15784_MOESM1_ESM.pdf]

Deep learning enables structured illumination microscopy with low light levels and enhanced speed

Jin et al.

Supplementary Table 1. The size of the datasets for different structures.

	adhesion	microtubule	mitochondria	F-actin
Training	669	749	953	882
Validation	99	167	135	162
Testing	32	204	61	85
Total	800	1120	1149	1129
Cells number	48	100	68	63

Supplementary Table 2. Quantitative estimation of the performance of U-Net-SIM.

	Structures	Average	U-Net-SIM3	U-Net-SIM15	Ground truth
PSNR	microtubules	18.27±2.68	32.31±5.12	35.75±5.44	x
	adhesions	18.69±2.88	24.84±1.89	29.48±1.61	x
	mitochondria	13.26±1.84	27.29±2.39	29.05±2.33	x
	F-actin	17.45±2.99	19.87±4.40	21.58±4.95	x
NRMSE	microtubules	1.05±0.21	0.27±0.12	0.19±0.10	x
	adhesions	0.73±0.25	0.47±0.10	0.28±0.06	x
	mitochondria	1.37±0.17	0.28±0.09	0.22±0.08	x
	F-actin	0.99±0.43	0.83±0.48	0.71±0.48	x
SSIM	microtubules	0.51±0.07	0.91±0.07	0.94±0.05	x
	adhesions	0.57±0.08	0.64±0.06	0.77±0.06	x
	mitochondria	0.30±0.10	0.79±0.06	0.83±0.05	x
	F-actin	0.38±0.14	0.54±0.15	0.63±0.18	x
RSP	microtubules	x	0.87±0.07	0.88±0.07	0.87±0.08
	adhesions	x	0.98±0.01	0.98±0.01	0.98±0.02
	mitochondria	x	0.41±0.25	0.42±0.25	0.43±0.24
	F-actin	x	0.94±0.05	0.95±0.05	0.92±0.07
RSE	microtubules	x	108.82±75.81	106.06±74.05	107.33±74.48
	adhesions	x	71.70±27.39	61.52±24.65	70.14±34.84
	mitochondria	x	389.08±256.07	384.19±253.90	384.52±252.95
	F-actin	x	120.16±60.42	107.45±68.59	122.39±60.30

Note: Source data and significance test are provided as a Source Data file. Data are presented as mean values +/- SD

Supplementary Table 3. Summary of imaging conditions.

		Exposure time	Laser power	Related main figures/vidoes	Related networks
Fixed cells	Normal light	200ms	10%	Fig 1; Fig 2;	U-Net-SIM15; U-Net-SIM3; U-Net-SNR
	Low light	20ms	1%	Fig 1; Fig 2;	U-Net-SIM15; U-Net-SIM3; U-Net-SNR
Live cells	Single-color Normal light	100ms	10%	Supp. Movie 1	U-Net-SIM15; scU-Nets
	Single-color Low light	5ms	1%	Fig. 3a; Supp. Movie 2	U-Net-SIM15; scU-Nets
	Dual-color Low light	50ms	2% for 488 nm; 1% for 561 nm	Fig. 3b; Supp. Movie 3	U-Net-SIM15; scU-Nets

Supplementary Table 4. Quantitative estimation of the performance of U-Net-SIM15 and scU-Net under low light condition.

	Structures	Average	Low light SIM	U-Net-SIM15	scU-Net	Ground truth
PSNR	microtubules	15.79±1.83	20.40±2.59	26.93±4.30	27.20±4.21	x
	adhesions	18.03±1.98	18.71±2.05	23.09±2.18	23.35±1.78	x
	mitochondria	20.32±1.95	20.32±2.54	23.67±1.92	23.75±1.94	x
	F-actin	16.95 ± 2.67	11.09 ± 7.64	19.18 ± 3.17	18.68 ± 3.89	x
NRMSE	microtubules	1.66±0.53	0.99±0.40	0.49±0.17	0.47±0.15	x
	adhesions	1.02±0.29	0.83±0.26	0.57±0.13	0.53±0.12	x
	mitochondria	0.63±0.14	0.60±0.16	0.40±0.09	0.40±0.10	x
	F-actin	1.06± 0.36	2.21 ± 1.38	0.84 ± 0.36	0.91 ± 0.48	x
SSIM	microtubules	0.19±0.08	0.43±0.14	0.83±0.12	0.83±0.12	x
	adhesions	0.23±0.06	0.33±0.08	0.55±0.09	0.55±0.08	x
	mitochondria	0.40±0.09	0.50±0.11	0.71±0.07	0.69±0.08	x
	F-actin	0.20 ± 0.09	0.38 ± 0.14	0.42 ± 0.16	0.44 ± 0.15	x
RSP	microtubules	x	0.60±0.10	0.63±0.14	0.63±0.13	0.62±0.14
	adhesions	x	0.74±0.09	0.84±0.07	0.85±0.06	0.83±0.07
	mitochondria	x	0.85±0.05	0.94±0.03	0.93±0.04	0.93±0.04
	F-actin	x	0.58±0.28	0.81±0.10	0.81±0.10	0.80±0.12
RSE	microtubules	x	1.67±0.68	1.56±0.59	1.54±0.56	1.55±0.57
	adhesions	x	1.75±0.42	1.35±0.21	1.34±0.21	1.39±0.22
	mitochondria	x	1.88±0.82	1.14±0.31	1.20±0.30	1.21±0.41
	F-actin	x	3.48±1.79	2.37±0.48	2.38±0.53	2.42±0.55

Note: Source data and significance test are provided as a Source Data file. Data are presented as mean values +/- SD

Supplementary Table 5. The achieved resolution of different approaches under low light conditions

	Average	SIM (Low light)	U-Net-SIM15	scU-Net	Ground truth
microtubules	865 ± 113	186 ± 2	170 ± 0.5	170 ± 0.6	169 ± 0.6
adhesions	545 ± 107	158 ± 1.5	156 ± 2	150 ± 1.7	150 ± 2
mitochondria	218 ± 17	256 ± 9	180 ± 4	179 ± 1.8	139 ± 3
F-actin	475 ± 20	179 ± 8	159 ± 2	179 ± 1.8	144 ± 1.2

unit: nm. Data are presented as mean values +/- SD. (Source data are provided as a Source Data file)

Supplementary figure 1: U-Net-SIM architecture.

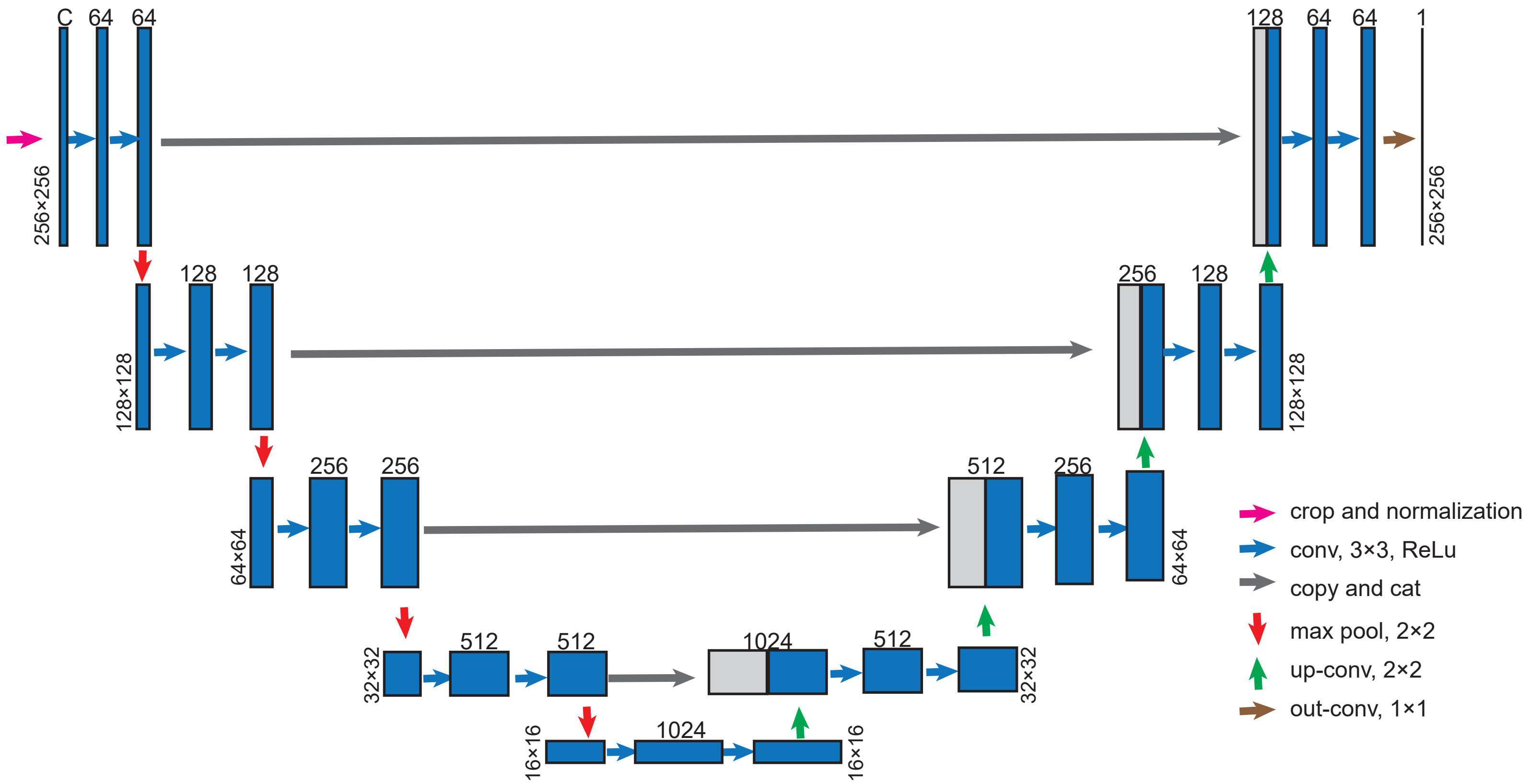

Supplementary Figure 1. U-Net-SIM architecture. C indicates the channel number of the input, which differs among different networks. U-Net-SIM15: C = 15; U-Net-SIM3: C = 3.

Supplementary figure 2: restoration error estimation of U-Net-SIM.

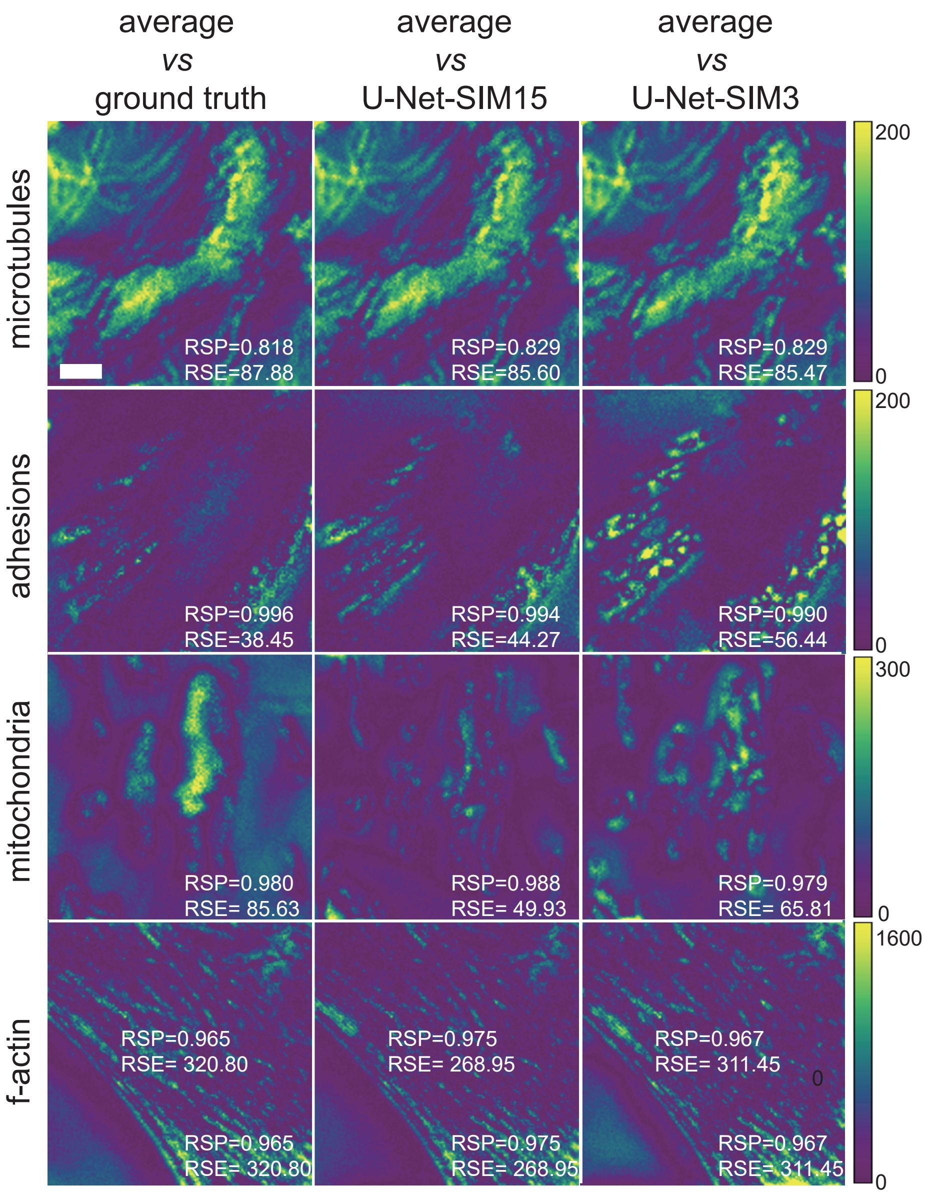

Supplementary Figure 2. Restoration error estimation of U-Net-SIM. The error maps were estimated via SQUIRREL for SIM reconstruction, U-Net-SIM15 and U-Net-SIM3 output against the average projection of the SIM raw data. Shown are the different structures addressed in Figure 1, which are randomly selected form the testing dataset indicated in Supplementary Table 1. The training datasets were collected from at least three independent experiments. Two metrics were calculated to quantify the restoration: the resolution-scaled error (RSE) and the resolution-scaled Pearson coefficient (RSP). Scale bar: 1 μm.

Supplementary figure 3

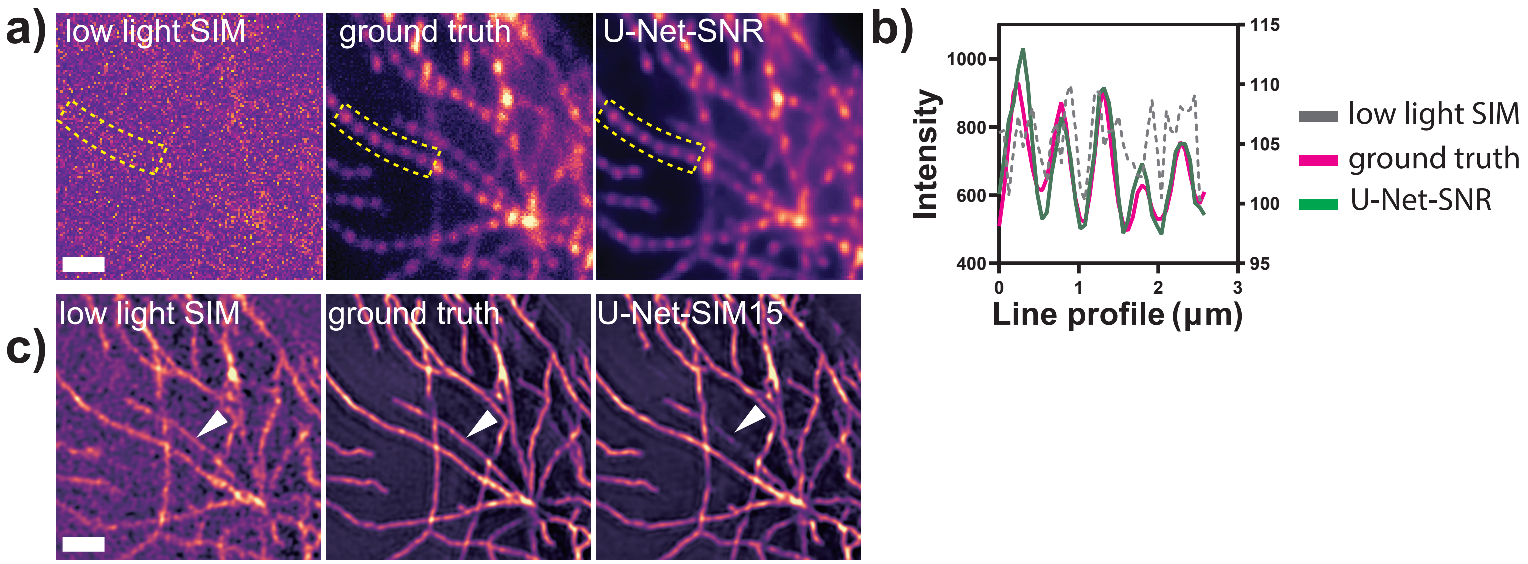

Supplementary Figure 3. Image recovery with U-Net-SNR and the pre-trained U-Net-SIM15. a, b) Microtubule samples were illuminated with structured patterns under low-light (**a**, left) and normal-light conditions (**a**, middle). U-Net-SNR were able to restore the structures (**a**, right), as well as the periodic illumination patterns along single microtubule as shown in **b**. In the line profile plot, low light SIM is shown on the right y-axis and all others share the left y-axis. One out of fifteen images are shown. Shown is a representative example randomly selected 204 testing samples. The training datasets were collected from at least three independent experiments. **c**) The output of U-Net-SNR were taken as the input of U-Net-SIM15 to retrieve SR information. Shown are SIM reconstruction results under low light conditions in the left panel of **c**, SIM reconstruction results under normal light conditions in the middle panel of c, and U-Net-SIM15 output taking images in the right panel of **a** as input. The combination of U-Net-SNR and U-Net-SIM15 achieves moderate restoration performance, but may fail at challenging areas, indicated by the white triangles. Scale bar: 1 µm.

Supplementary figure 4: scU-Net architecture.

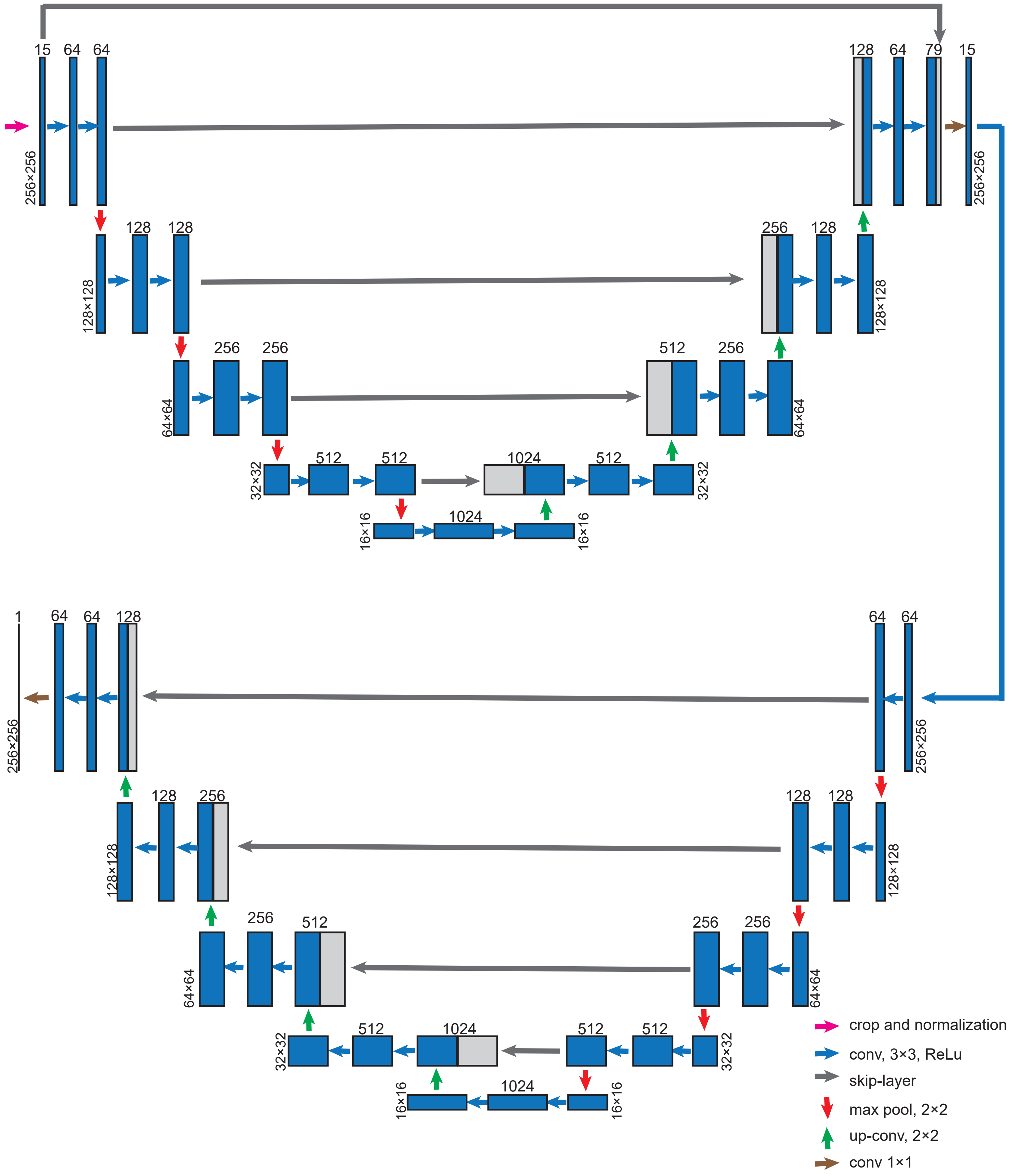

Supplementary Figure 4. scU-Net architecture. Two U-Nets were stacked together via skip-layer connection. The network was trained by taking 15 SIM raw images under low light conditions as the input and the SIM reconstruction under normal light conditions as the ground truth.

Supplementary figure 5: restoration error estimation of scU-Net.

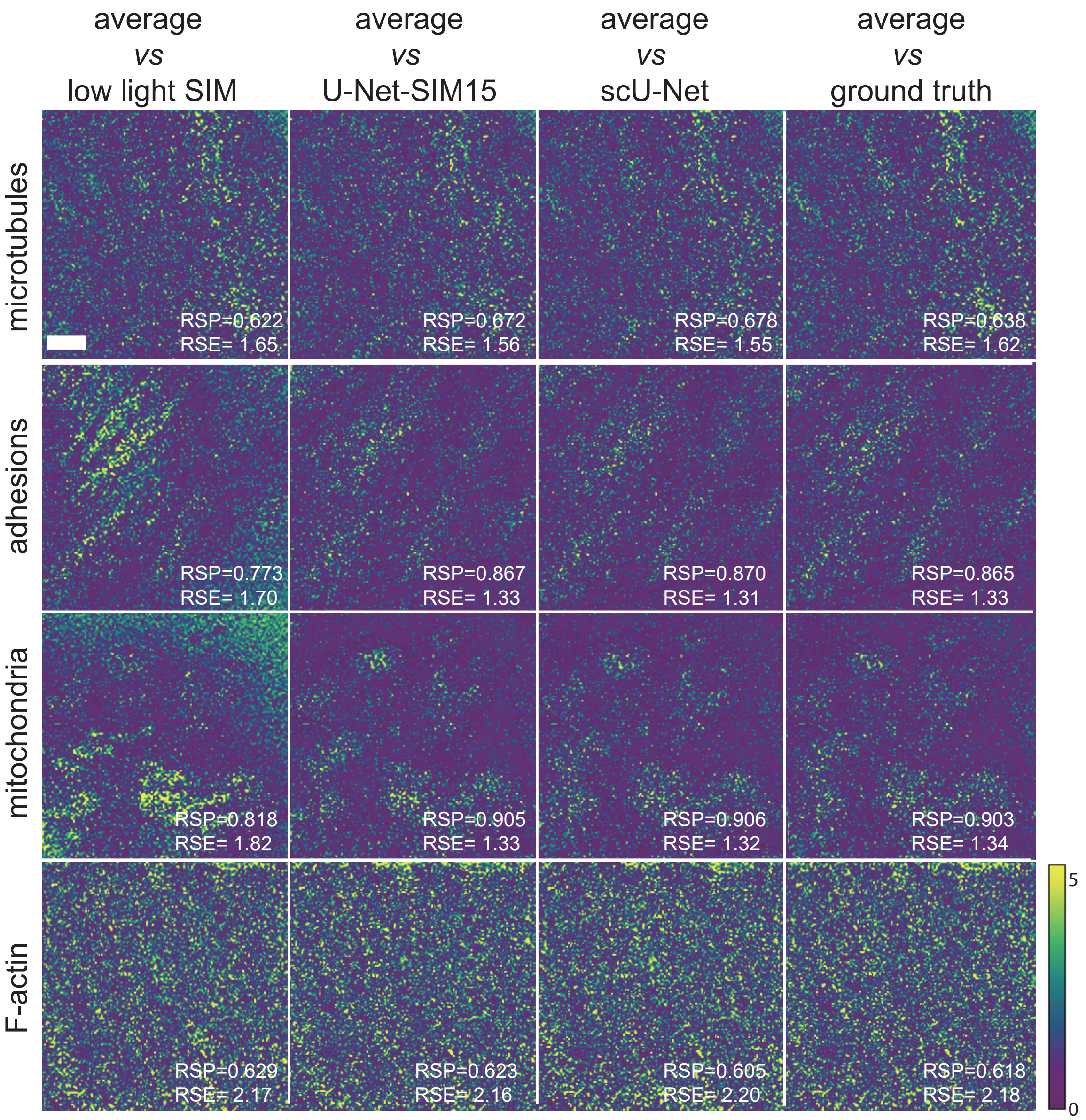

Supplementary Figure 5. Restoration error estimation of scU-Net. The error maps were estimated via SQUIRREL for the indicated restoration approaches. Shown are the different structures addressed in Figure 2, which are randomly selected form the testing dataset indicated in Supplementary Table 1. The training datasets were collected from at least three independent experiments. The values of RSE and RSP were calculated to quantify the restoration. Scale bar: 1 μm.

Supplementary figure 6: photobleaching of SIM imaging under low light and normal light conditions.

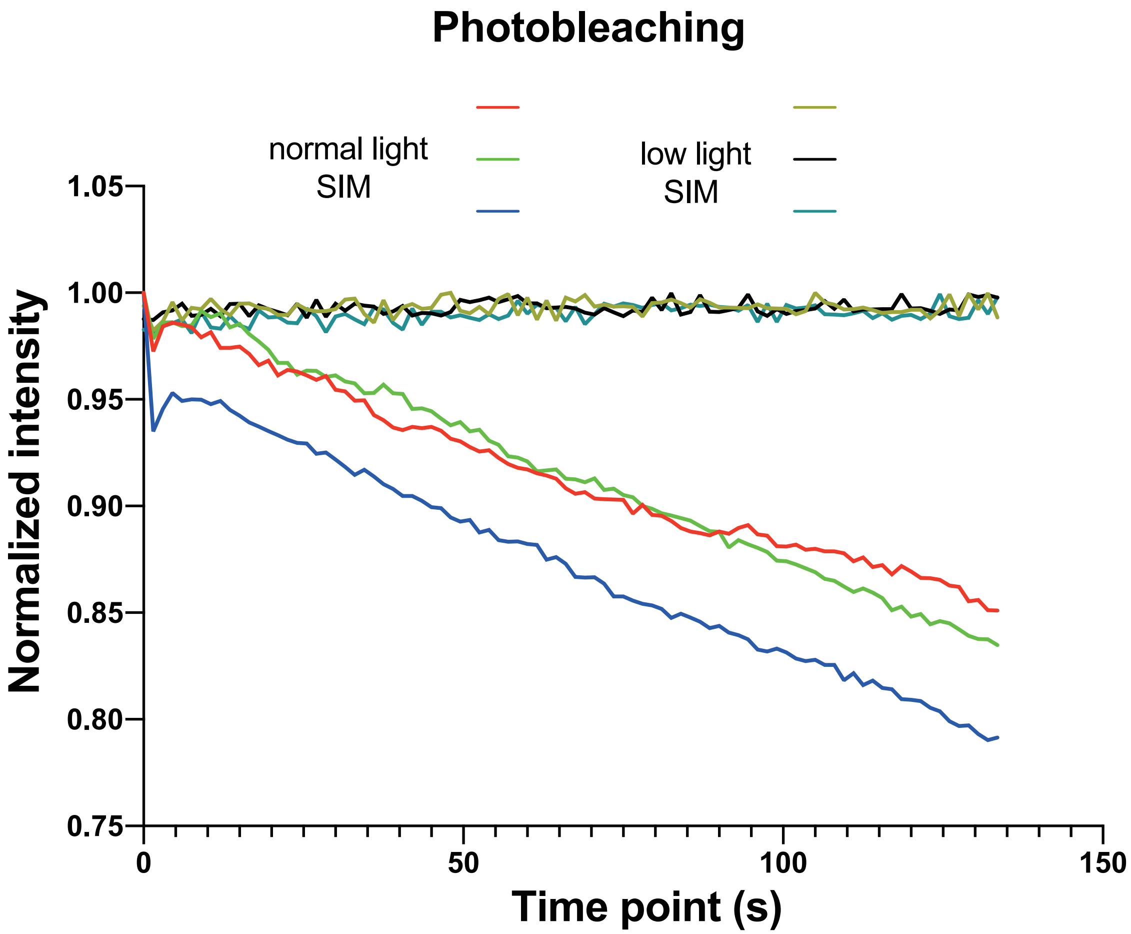

Supplementary Figure 6. Photobleaching of SIM imaging under low light and normal light conditions. COS-7 cells were transiently transfected with EMTB-3XmCherry. For normal light SIM imaging, we used 10% 561 nm laser power and 100 ms exposure time, while for the low light SIM imaging, we used 1% 561 nm laser power and 10 ms exposure time. Shown are different cells in a single experiment.

Supplementary figure 7

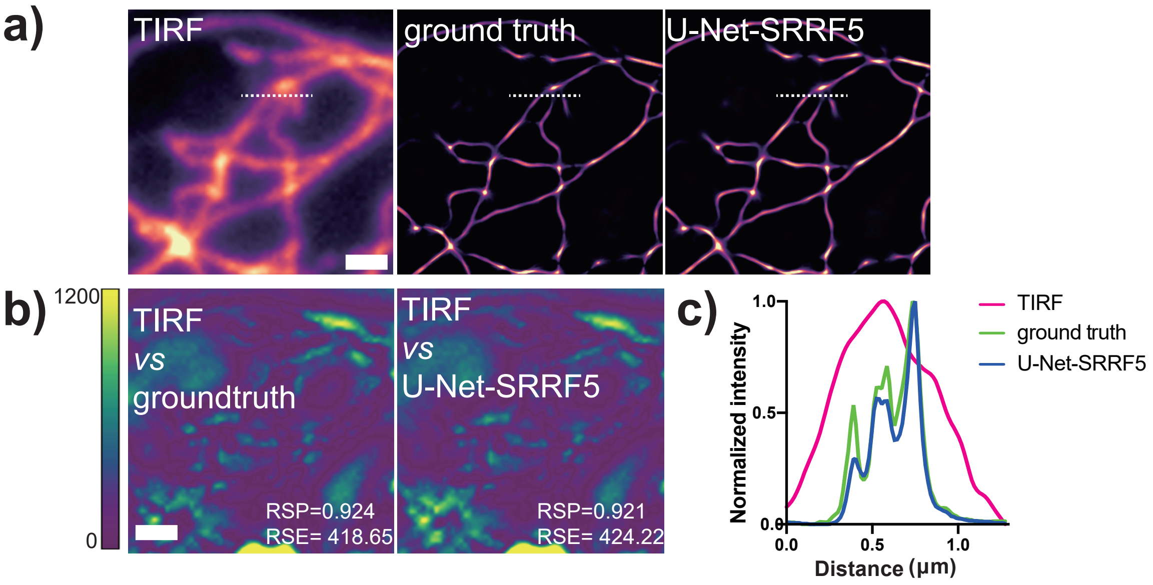

Supplementary Figure 7. U-Net-SRRF5 restoration. MEF cells were fixed and the microtubules were stained with Alexa-647 dye. The cells were examined under a TIRF microscope. **a)** Shown are: a representative TIRF image of microtubules (**left**), SRRF reconstruction of 200 TIRF images, output of U-Net-SRRF5 by taking five TIRF images as input. **b**) Restoration error of SRRF and U-Net-SRRF. **c**) Line profiles along dotted lines in **a** show resolution improvement of U-Net-SRRF5. The value of RSE and RSP were calculated to quantify the restoration. Scale bar: 1 μm.

Supplementary figure 8

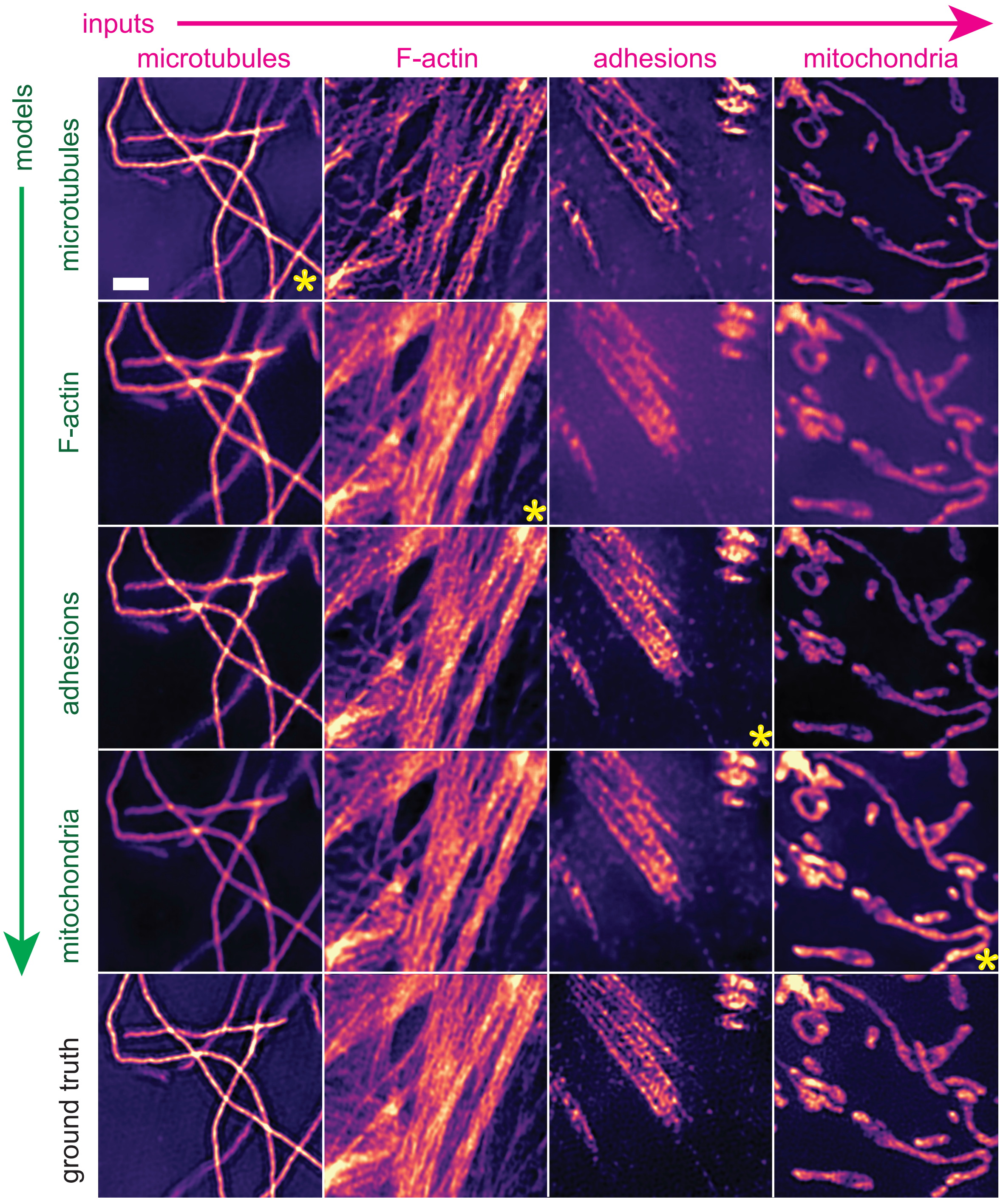

Supplementary Figure 8. Apply a model trained on one structure to different structures. Each column shows input datasets of indicated structures. Each row shows the selected model pre-trained with the indicated structures and the last row shows the SIM reconstruction of the input dataset. The panels marked with a yellow asterisk were tested using the correct model. The dataset is from at least three independent experiments and the exact number of each dataset is indicated in Supplementary Table 1. Scale bar: 1 µm.

Supplementary figure 9

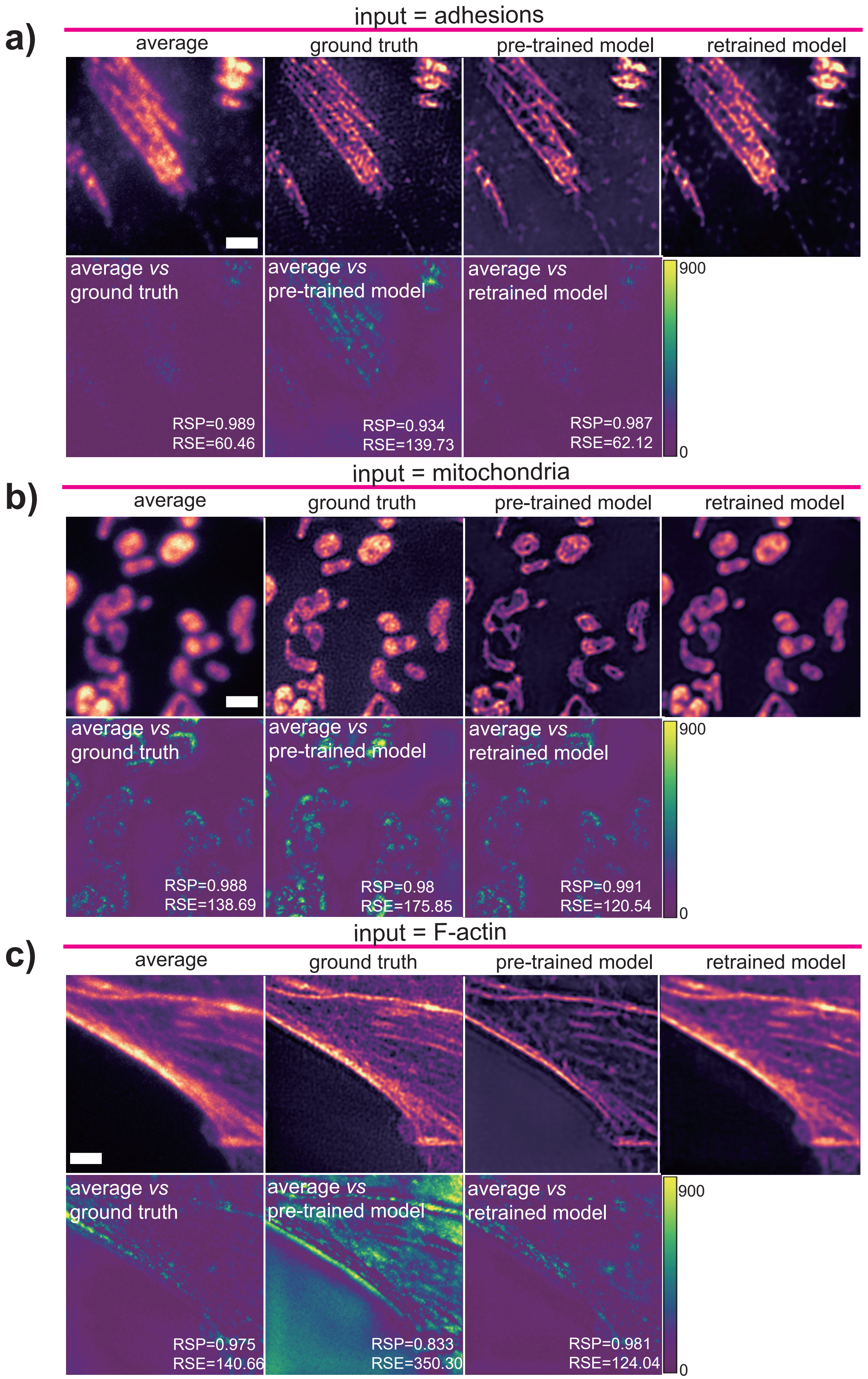

Supplementary Figure 9. Transfer learning. To illustrate transfer learning, we made use of the network pre-trained on microtubules samples to initiate a new network and retrained it on other structures: adhesions (**a**), mitochondria (**b**) and F-actin (**c**). Fifteen SIM raw images were taken as the input. For each panel, top row: the average projection, the SIM reconstruction (ground truth), the output of the pre-trained model, and the output of re-trained model. The restoration error maps were estimated via SQUIRREL. The second row: SIM reconstruction, output of the pre-trained model and output of the retrained model. The value of RSE and RSP were calculated to quantify the restoration. The dataset is from at least three independent experiments and the exact number of each dataset is indicated in Supplementary Table 1. Scale bar: 1 μm.
