# Supplementary material for: Deep learning enables structured illumination microscopy with low light levels and enhanced speed
Source: Nat Commun. 2020 Apr 22;11:1934. doi: 10.1038/s41467-020-15784-x (PMC7176720; doi:10.1038/s41467-020-15784-x)
Supplement: Supplementary file 3 — Description of Additional Supplementary Files [file 41467_2020_15784_MOESM3_ESM.pdf]

Description of Additional Supplementary Files

File Name: Supplementary Movie 1
Description: **SR imaging of microtubules in living cells.** Shown are: the average projection of fifteen SIM raw images, SIM reconstruction, output of U-Net-SIM15 and output of U-Net-SIM3. Raw data was collected under normal light conditions (10% of 561 nm laser and 100 ms exposure time). Scale bar: 1 µm.

File Name: Supplementary Movie 2
Description: **SR imaging of microtubules under low light conditions**. Shown are: the average projection of fifteen SIM raw images, SIM reconstruction, output of U-Net-SIM15 and output of scU-Net. Raw data was collected under low light conditions (1% of 561 nm laser and 5 ms exposure time). Scale bar: 1 µm.

File Name: Supplementary Movie 3
Description: **Dual-color imaging under low light conditions with scU-Net.** COS-7 cells were transfected with EMTB-3XmCherry overnight and stained with MitoTracker Green before imaging **(Methods).** Shown are: SIM reconstruction (left) and output of scU-Net (right). The data was collected using 2% of 488 nm laser and 1% of 561 nm laser with 50 ms exposure time. Scale bar: 1 µm.
